# Mechanical Behavior and Durability Performance of Concrete Reinforced with Hybrid Date Palm and Polypropylene Polymer Fibers

**DOI:** 10.3390/polym17101350

**Published:** 2025-05-15

**Authors:** Musa Adamu, Wafa Abdelmajeed Labib, Yasser E. Ibrahim, Hani Alanazi

**Affiliations:** 1 Engineering Management Department, College of Engineering, Prince Sultan University, Riyadh 11586, Saudi Arabia; ymansour@psu.edu.sa; 2 Structures and Materials Research Laboratory, College of Engineering, Prince Sultan University, Riyadh 11586, Saudi Arabia; 3 Architecture Department, College of Architecture and Design, Prince Sultan University, Riyadh 11586, Saudi Arabia; wlabib@psu.edu.sa; 4 Department of Civil and Environmental Engineering, College of Engineering, Majmaah University, Al-Majmaah 11952, Saudi Arabia

**Keywords:** cementitious composite, natural fibers, date palm fiber, polypropylene polymer fiber, hybridization, interfacial transition zone, fiber–cement matrix

## Abstract

Concrete faces challenges related to brittleness and crack propagation, which compromise its tensile strength and durability. Fiber reinforcement has emerged as a promising solution, yet research on hybrid systems combining natural fibers, such as date palm fiber (DPF), with synthetic polymer fibers, like polypropylene fiber (PPF), remains limited. This study investigates the mechanical and durability performance of concrete reinforced with hybrid DPF and PPF, aiming to address the gap in understanding the synergistic effects of combining natural and synthetic fibers in cementitious materials, and improving the tensile strength and crack resistance of the concrete. Both the DPF and PPF were added at varying dosages (0%, 0.25%, 0.5%, 0.75%, and 1% by weight of cement). Both DPF and PPF reduced the workability, fresh density and compressive strength of concrete, with DPF exhibiting a more significant reduction due to its higher hydrophilicity and poor compatibility with the cement matrix. A maximum reduction of 44.78% was observed in the mix containing 1% DPF and 0.5% PPF. The fibers improved tensile strength and ductility, with mixes containing up to 1% combinations of DPF and PPF showing up to a 14.6% increase in splitting tensile strength and 9.5% improvement in flexural strength compared to the control mix. However, durability was compromised—water absorption increased by up to 58% in hybrid mixes containing 1.5% total fiber content, while pore volume rose by as much as 17.5% compared to plain concrete. These increases were more pronounced with higher DPF content due to its hydrophilic nature and poor cement compatibility. This study highlights the potential of hybrid fibers to improve concrete performance while promoting eco-friendly and cost-effective solutions.

## 1. Introduction

Concrete is the most universally used construction material due to its availability, affordability, and superior compressive strength. However, it is intrinsically prone to cracking and exhibits minimal tensile resistance and flexural strength, which can lead to crack formation and reduced durability [1,2]. To address these limitations, fiber reinforcement has been explored as an effective solution to enhance the crack resistance and tensile strength of the concrete. Several studies have proven that fiber addition enhances tensile and flexural strengths, impact resistance, and fracture toughness while also improving fatigue and bond strength [3,4,5,6,7]. Both natural and synthetic fibers have been investigated for their role in reinforcing concrete [8,9]. However, a comparable assessment of how adding natural and synthetic fibers can be beneficial in determining its strength-to-cost ratio [10,11,12]. Natural fibers, such as date palm fiber (DPF), are abundant, eco-friendly, and cost-effective, making them attractive for sustainable construction [11]. However, their use in concrete has presented challenges, including increased water absorption, reduced compressive strength, and potential durability concerns due to their hydrophilic nature [13]. On the other hand, synthetic fibers, such as polypropylene fiber (PPF), are widely used due to their superior tensile strength, low density, and resistance to chemical degradation [14]. PPF has been found to enhance ductility, flexural toughness, and crack resistance, though excessive use may lead to fiber agglomeration and reduced bond strength within the concrete matrix [4,15]. While several studies have examined the effects of DPF and PPF individually in concrete [16,17,18,19], research on their combined effects in a hybrid fiber-reinforced system remains limited. Given that both fiber types have distinct advantages and limitations, hybridization could optimize concrete performance by leveraging their complementary properties. For instance, while DPF may enhance sustainability and toughness, PPF could improve tensile strength and durability, mitigating some of the drawbacks associated with natural fibers [11,15,16,20,21]. However, due to variations in fiber properties, their combined effect on mechanical and durability characteristics requires further investigation.

Different types of natural fibers have been incorporated to improve the properties of cement composites. Razmi and Mirsayar [22] found that adding 0.1% jute fiber by weight of cementitious materials improved fracture toughness by up to 45% and enhanced compressive, flexural, and splitting tensile strengths by 10–37%, 5–10%, and 5–17%, respectively. Hasan, Sobuz [23] reported enhancement in compressive strength by up to 2%, increasing tensile strength by up to 21%, and flexural strength by up to 18% by adding 0.25% jute fiber by volume, while also improving ductility and post-cracking behavior due to effective fiber bridging. Veerappan, Mani [24] added jute and coir fibers separately in concrete. They found that adding 0.5% jute fiber by volume improved the compressive strength by up to 19.30% and flexural strength by up to 16.56% in M-sand concrete, along with better post-cracking behavior due to effective fiber bridging. Thus, the coir fiber outperformed the jute in terms of improvements on the concrete’s properties. Nawab, Ali [25] found that incorporating 6% coconut fiber by weight of cementitious materials in mortar, along with 10% silica fume and 10% metakaolin, enhanced compressive strength by up to 28.3% and flexural strength by up to 16.7 MPa after 28 days. Vélez, Rodríguez [26] reported enhancements in compressive strength of concrete with the addition of 0.1% and 1% coconut fibers and treatment of the fiber with NaOH leading to better improvement. According to the findings of Kiamahalleh, Gholampour [27], incorporating 3% sugarcane fiber by fine aggregate mass enhanced compressive strength by 16%, flexural strength by 35%, and reduced water absorption and chloride ion penetration by 4% and 23%, respectively, while further increases in fiber content decreased performance. Abdulkareem, Ayeronfe [28] found that incorporating 0.2% kenaf fiber in bio-concrete increased compressive strength by 12% and flexural strength by 60%, while higher fiber content reduced compressive strength but improved flexural performance, with optimal flexural strength observed at 0.5% kenaf fiber in recycled aggregate concrete. Antwi-Afari, Mutuku [29] reported improvement in the compressive strength of concrete by up to 11.7% and tensile strength by up to 12.6%, with addition of up to 2% treated sisal fiber.

Polymer fibers have also been used to improve the performance of concrete. Wang, Qiao [30] found that incorporating polyacrylonitrile fiber (PANF) at 0.8 kg/m^3^ improved the compressive strength of recycled concrete by up to 36.6% and enhanced frost durability. Zhao, Cai [31] incorporated polyethylene (PE) fibers into concrete, and observed improved tensile strain capacity and energy absorption, with PE fiber (15 mm length, 27 µm diameter, 117 GPa modulus) showing optimal performance, achieving an ultimate tensile strain of 1.155% and significantly enhancing tensile toughness. Flores Nicolás, Menchaca Campos [32] reported that incorporating 0.2% and 0.4% HDPE fibers by weight of sand slightly improved flexural strength (up to 2.3%) and compressive strength (4.8% for short fibers), while also reducing corrosion rates in reinforced concrete exposed to saline environments. Sridhar, Kumar [33] found that incorporating 0.5–1.5% flattened-end nylon fiber (FENF) by volume improved split-tensile and flexural strengths by up to 25.1% and 26.1%, respectively, compared to conventional concrete. The study also reported that FENF enhanced compressive strength by up to 10.3%. Ahmad, Zaid [34] added 1.5% nylon fiber by weight of cement and reported improved compressive strength by up to 24% and enhanced splitting tensile strength by up to 47%, compared to the control mix. They also observed that durability properties, such as water absorption, acid resistance, carbonation depth, and permeability, were significantly improved with the incorporation of nylon fibers. Chandrasekhar, Ransinchung RN [35] found that incorporating a hybrid blend of PVA and polyester fibers in engineered cementitious composites (ECCs) improved compressive strength by 18–30% when river sand was partially or fully replaced with manufactured sand. Additionally, the study revealed that while fiber blending slightly reduced slump flow (1.3–4.5%), it significantly enhanced durability properties, including reduced drying shrinkage due to improved matrix–aggregate bonding and fiber–matrix interaction.

Despite several advantages, DPF and PPF present challenges when incorporated into concrete. DPF addition tends to reduce mechanical properties, including compressive strength and elastic modulus [12,36,37]. It also negatively affects durability by increasing water absorption, drying shrinkage, and porosity [11,12]. These issues primarily arise due to the hydrophilic nature of DPF, poor fiber–cement adhesion, and the retention of water within the fiber pores, which weakens the cement matrix [11,12]. Similarly, PPF addition can lead to fiber agglomeration, which disrupts the bond between concrete ingredients, potentially weakening the overall matrix [38]. Moreover, while PPF improves toughness and ductility, it is less effective in reducing shrinkage due to its rough surface texture [39]. PPF exhibits higher capillary water absorption and increased porosity [39,40]. However, these limitations can be mitigated through hybridization, where combining DPF and PPF in optimized proportions may balance their strengths and weaknesses [39].

Osman, ALyamany [41] examined the effects of hybrid DPF-PPF reinforcement on fresh and hardened concrete. The findings showed that adding both fibers reduced the concrete compacting factor but enhanced flexural strength. Adjusting fiber proportions makes it possible to achieve optimal mechanical performance. Compared to DPF, PPF exhibits a higher elongation at break [42], indicating superior ductility and flexibility, making it more suitable for applications requiring resistance to deformation. In terms of mechanical properties, DPF has a diameter of 100–1000 µm, tensile strength of 58–203 MPa, flexural strength of 76–117 GPa, compressive strength of 60–70 MPa, Young’s modulus of 2–7.5 GPa, and break elongation of 5–10% [43,44]. In contrast, PPF exhibits a diameter of 20–25 µm, tensile strength of 350 MPa, density of 910 kg/m^3^, flexural strength of 31.6 GPa, compressive strength of 538.6 MPa, Young’s modulus of 3 GPa, and break elongation of 120% or higher [39,45].

Concrete performance is often assessed based on mechanical and durability properties. Mechanical properties include compressive strength, flexural strength, splitting tensile strength, ductility, toughness, and drying shrinkage strain [7,11,13], while durability properties include permeability, capillary absorption, resistivity, density, porosity, leaching resistance, and carbonation depth [46,47]. Existing literature suggests that DPF and PPF individually contribute to increased tensile strength, flexural toughness, and ductility while reducing concrete density. However, their combined effects require further investigation to optimize their hybridization for enhanced mechanical and durability performance. While numerous studies have explored combinations such as steel–polypropylene or glass–polypropylene fibers, the hybrid use of DPF and PPF has only recently begun to be investigated. The DPF is unique in terms of its morphology, hydrophilicity, and regional availability, especially in arid regions like Saudi Arabia. This makes it an attractive candidate for sustainable construction when combined with PPF, which helps mitigate some of DPF’s limitations (e.g., poor water resistance and low ductility). Unlike many prior studies that focus on single-dosage comparisons, in this study a comprehensive evaluation across multiple dosage levels (0%, 0.25%, 0.5%, 0.75%, and 1% by weight of cement) of both individual and hybrid fiber systems was conducted. This allows the determination of the optimal balance between workability, mechanical performance, durability, and microstructural behavior of the hybrid DPF-PPF-reinforced concrete.

Thus, the goal of this research is to evaluate the mechanical and durability performance of hybrid DPF-PPF-reinforced concrete. The research assesses key properties such as compressive strength, tensile strength, flexural strength, toughness, water absorption, and porosity. The study also explores the challenges associated with fiber hybridization and the potential trade-offs between mechanical performance and long-term durability. This research contributes to developing sustainable and high-performance fiber-reinforced concrete by optimizing fiber dosage and understanding their combined behavior.

## 2. Materials and Methods

### 2.1. Materials

**Figure 1:**
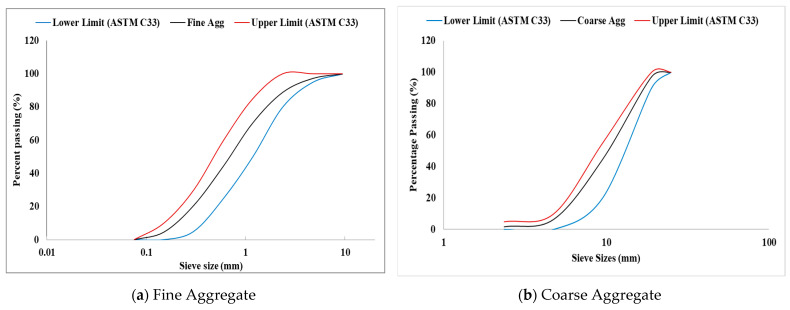
Particle gradation of aggregates.

This study utilized Ordinary Portland Cement (OPC), Type I, as the cementing material. The OPC’s properties are given in Table 1, where it conformed with the specifications of ASTM C150 [48]. The fine aggregate utilized was a well-graded natural river sand, and its particle gradation is shown in Figure 1a. The aggregate has a specific gravity of 2.65, a bulk density of 1563 kg/m^3^, and water absorption of 1.77%. The coarse aggregate was crushed gravels obtained from a nearby quarry. Based on the particle size gradation in Figure 1b, the coarse aggregate was categorized as well-graded and recommended for use in concrete based on ASTM C33/C33M [49] specifications. The coarse aggregate exhibited a nominal maximum size of 19 mm, bulk density of 1458 kg/m^3^s specific gravity of 2.66, and water absorption value of 0.78%. A polycarboxylate-based superplasticizer, with a density of 1060 kg/m^3^, was incorporated as a water-reducing additive in the mixtures.

**Figure 2:**
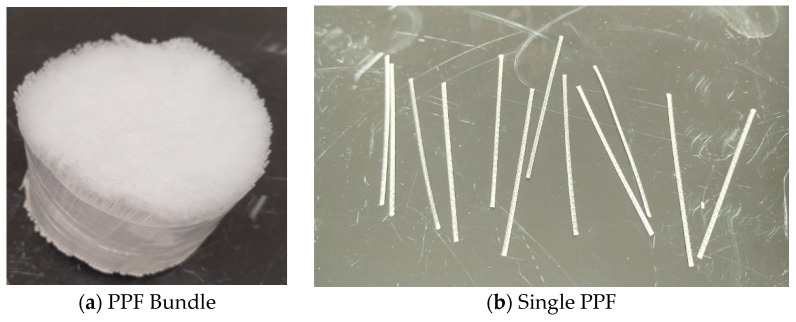
Polypropylene polymer fiber used.

This study used two types of fiber: natural fiber, date fiber (DPF), and synthetic fiber, which is a coarse type of polypropylene fiber (PPF). A commercially available MasterFiber 142 obtained from MBS Construction Chemicals LLC, Dubai, UAE, that conforms to the standard specifications of both BS EN 14889-2 [50] and ASTM C1116 [51] was used as the PPF. The properties of the PPF, as obtained from the manufacturer, are summarized in Table 2, and its morphology is shown in Figure 2.

**Figure 3:**
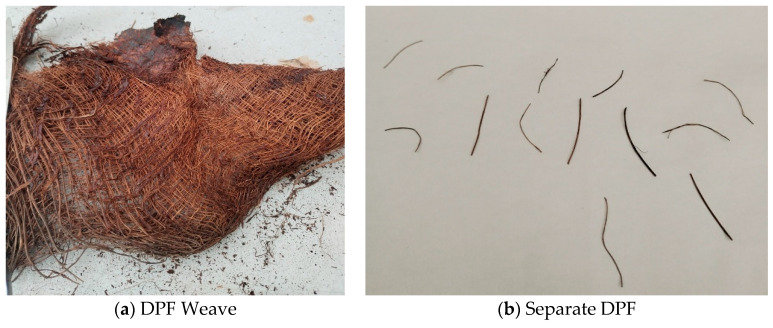
Date palm fiber used.

The DPF was obtained by processing the date tree fiber mesh. The fiber mesh was collected from a neighboring date palm tree farm in the outskirts of Riyadh, Saudi Arabia, in the form of an interwoven mesh with lengths ranging from 30 to 50 cm and width from 20 to 30 cm, as shown in Figure 3a. The fiber mesh was first submerged in water for about 2 h and then thoroughly washed. After washing, it was treated using a 3% NaOH solution to remove impurities further and enhance the fiber’s roughness. The fiber mesh was soaked in the NaOH solution for 120 min and then thoroughly washed with water. During washing and treatment, the fiber mesh was disentangled into single fibers. After washing and treatment, the single DPF was kept in open air until completely dried. The single DPFs were 20–30 mm long with diameters between 0.2 and 1.0 mm. Figure 3b shows the morphology of the single-treated DPFs, and their properties are highlighted in Table 2.

### 2.2. Mix Proportioning

The standard procedure of ACI 211.1R [52] was employed for designing the control concrete mix in this study. The DPF and PPF were added to the concrete mixes in several dosages of 0%, 0.25%, 0.5%, 0.75%, and 1% by weight of cement. The superplasticizer was added in constant proportion to all the mixes, at a concentration of 1% relative to the cement’s weight. A total of ten (10) mixes including the control mix were generated based on the different proportions of the DPF and PPF, as shown in Table 3. For simplicity of comprehension, each mix was given a distinct identification code based on the proportion of DPF and PPF present in the mixture. The mix labeled M0D0P represents the reference mix, containing no DPF (0%) and no PPF (0%). Meanwhile, M0.25D0.5P refers to the blend that includes 0.25% DPF and 0.5% PPF, and M0.5D1P denotes the mixture composed of 0.5% DPF and 1% PPF.

### 2.3. Sample Preparation

The concrete batching, mixing, and curing processes were carried out in accordance with the standard procedures outlined in ASTM C192 [53]. A revolving drum concrete mixer was utilized for mixing the concrete. All the constituent materials were weighed and prepared in advance of mixing. It was confirmed that the DPF and PPF showed no signs of agglomeration before mixing. The water and superplasticizer were stirred together in a single container and then divided into two equal parts before the mixing began. The fine materials, i.e., cement, and fine aggregates were placed into the mixer at the start and mixed until completely homogenized. Next, the DPF, PPF, coarse aggregates, and half of water plus superplasticizer were poured in and mixed for around a minute. While the mixing was ongoing, the second portion of the water and superplasticizer was steadily introduced into the mixer. The concrete was allowed to mix properly in the mixer until an entirely homogeneous mix was achieved, and the fibers were well dispersed within the fresh concrete without any balling effect. Subsequently, the workability and density of the freshly mixed concrete were measured after mixing. Soon afterward, the fresh concrete was put into the prepared molds and allowed to harden by keeping in the laboratory for 24 hrs. After hardening, the concrete samples were extracted from the molds and cured in water. The concrete samples were kept in the water until the designated day of testing.

### 2.4. Testing Methods

The properties of the concrete measured at fresh state include workability and fresh density. The slump test in accordance with ASTM C143/143M [54] specifications was used to evaluate the workability of the concrete. The fresh density was tested in accordance with ASTM C138/C138M [55] specifications. After curing the 100 mm cubic specimens in water for 7 and 28 days, the compressive strength of the hardened concrete was tested using the methods described in BS EN 12390-3 [56]. After undergoing curing for a period of 7 and 28 days, the cylindrical concrete samples with a height of 200 mm and diameter of 100 mm were tested for split tensile strengths with reference to the specifications of BS EN 12390-6 [57]. For the flexural strength, prismatic concrete samples with dimensions of 100 × 100 × 500 mm were cured for a period of 7 and 28 days and then tested as per the guidelines of ASTM C293 [58]. The concrete’s water absorption was measured according to the methods illustrated in ASTM C642 [59] using cubic samples with 100 mm dimensions, which were cured for 28 days in water before testing. Triplicate specimens were prepared and tested for all the mixes and each curing period, and the mean result was recorded.

Microstructural characterization, including mercury intrusion porosimetry (MIP) and scanning electron microscopy (SEM), were performed on selected concrete mixes. The representative mixes selected exhibited distinct mechanical and durability behavior, including the control mix (M0D0P), single-fiber mixes (M0.5D0P and M0D0.5P), and hybrid mixes (M0.5D0.5P, M0.5D1P, and M1D0.5P). These selections were made to capture the effects of fiber type and combination on microstructure and pore structure without excessive repetition. Small concrete pieces were extracted from the specimens after 28 days of strength testing, dried, and used for the SEM and MIP tests. A high-pressure vacuum was used to eliminate impurities from the surface of the concrete fragments. The SEM test was performed using the standard procedures of ASTM C1723 [60]; high-resolution images of the concrete microstructure were captured at different resolutions of up to 10,000 magnifications, and the best images were selected and reported. The MIP test was performed by adopting the standard methods listed in ASTM D4284 [61], using a Scientific Porosimeter. The samples’ pore volumes, diameter, and porosity were extracted from the computer output system attached to the porosimeter.

## 3. Results and Discussion

### 3.1. Workability

**Figure 4:**
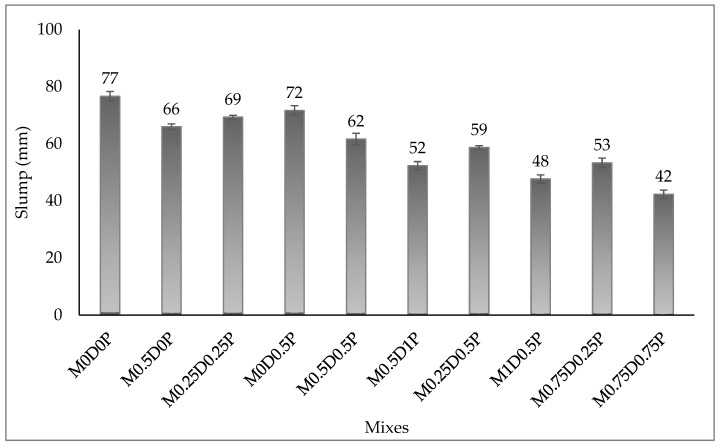
Slump test results.

The slump test was used to measure the workability of the hybrid fiber-reinforced concrete mixes, and the results are shown in Figure 4. Both DPF and PPF contributed to a decline in the workability of the concrete mixes. For proper illustration, compared to the slump value of 77 mm for the reference mix (M0D0P), the slump of mix M0.5D0P was 66 mm. Likewise, the slump value for mix M0D0.5P was 72 mm, lower than that for mix M0D0P. The loss of workability in concrete due to adding DPF was attributed to its strong hydrophobicity and high concentration of cellulose and hemicellulose This makes the DPF highly wettable during mixing, where it can absorb a high proportion of water during mixing the concrete, thereby reducing the amount of free water available for mixing and achieving consistency in the mix, which reduces the consistency of the concrete mix. Similar results have been reported by both Ibrahim, Adamu [11] and Ali-Boucetta, Ayat [16]. The reduction in slump value of the concrete with the addition of PPF might have resulted from the retention and confinement effects of the PPFs [17]. Another reason might be the formation of a network structure in the concrete with the addition of the PPF, where the fiber attracts some cement paste across its surface; this increases the stiffness of the fresh concrete, making it more viscous and reducing its slump value [15]. Previous studies by Qin, Zhang [17], Leong, Mo [20], and Bentegri, Boukendakdji [18] have reported a reduction in the workability of concrete with the addition of PPF. By comparing the slump values of the concrete mixes in Figure 4, the DPF was found responsible for a greater reduction in the slump/workability of the concrete mixes compared to PPF. This can be clarified by assessing the slump values of the mixes with the same proportion of DPF or PPF. Mix M05D0P with only 0.5% DPF has a slump value of 66 mm, while mix M0D0.5P with only 0.5% PPF has a higher slump value of 72 mm. Similarly, hybrid DPF-PPF-reinforced concrete mixes containing higher DPF and lower PPF exhibit lower slump than mixes with lower DPF and higher PPF. Mix M1D0.5P containing 1% DPF and 0.5% PPF has a lower slump value (48 mm) than mix M0.5D1P with 0.5% DPF and 1% PPF with a slump of 52 mm. This happens because the DPF is a vegetable fiber with high hemicellulose and hydrophilicity, making it more wet and absorbing more water during mixing than the PPF [62]. Acosta-Calderon et al. [51] reported a similar observation, where they found that adding sisal fibers to concrete caused a higher reduction in slump values compared to when PPF was added.

### 3.2. Fresh Density

**Figure 5:**
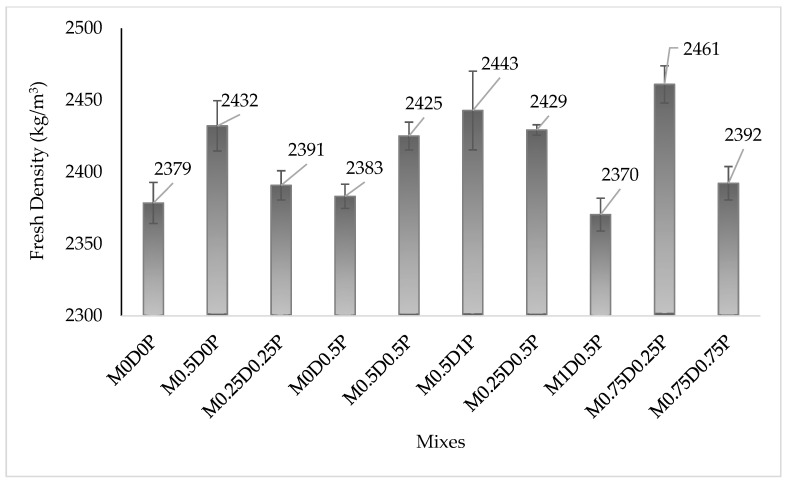
Results of fresh density.

The results of the fresh density (unit weight) of the concrete mixes containing different proportions of hybrid DPF and PPF are presented in Figure 5. Adding both DPF and PPF led to a slight decline in the fresh density of the concrete. However, DPF was found to have a more pronounced negative effect on unit weight than PPF. This can be verified by comparing the fresh density values of mix M0.5D0P and M0D0.5P with that of mix M0D0P (Control). Mix M0.5D0P has a reduction in fresh density by 2.07% about mix M0D0P, while the fresh density of mix M0D0.5P was lower by 1.34% compared to that of mix M0D0P. The reasons for the reduction in density with the addition of fibers (DPF and PPF) to the concrete may be as follows: the fibers have lower density compared to the cementitious materials [19] and fibers entrap air on their surface during mixing, increasing the air content and voids in the concrete mix, leading to lower unit weight [11,63]. Studies by Qin, Zhang [17] and Yong, Yew [19] reported a slight reduction in the fresh density of concrete with the addition of PPF, which they also ascribed to the lower density of the PPF. Ibrahim et al. [11] also reported a slight reduction in the fresh density of concrete with the addition of DPF. The addition of hybrid DPF and PPF causes a further reduction in the density, as shown in Figure 5, due to the combined effects of both DPF and PPF on the unit weight of the concrete. Mixes containing higher DPF and lower PPF exhibit lower density than those with lower DPF and higher PPF. In addition, mix M1D0.5P has a lower density value (2270 kg/m^3^), while mix M0.5D1P has a higher density value (2296 kg/m^3^). DPF is more porous, wettable, and has lower bulk density than PPF. This makes the DPF entrap more air, creates more pores, and causes a decline in the unit weight of the concrete compared to PPF. These lower unit weights of the hybrid fiber-reinforced concrete are beneficial for its use as lightweight concrete.

### 3.3. Compressive Strength

**Figure 6:**
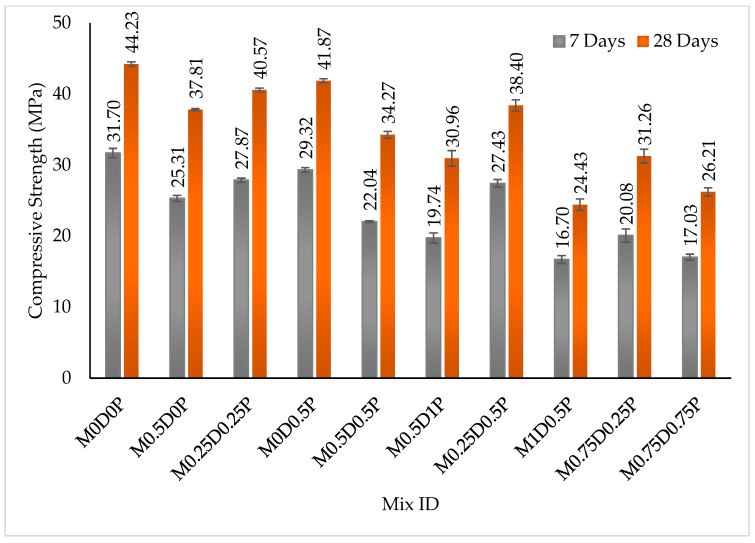
Results of compressive strength.

Figure 6 provides a summary of the compressive strength results for the DPF-PPF hybrid fiber-reinforced concrete mixes. Through the continuous hydration reaction, the compressive strength increases with the age of curing. There was a reduction in the compressive strengths at 7 and 28 days of curing with the addition of DPF and PPF. Adding 0.5% DPF only to the concrete mixes lowered the compressive strength. The strength of the mix with 0.5% DPF (M0.5D0P) was less than the control mix (M0D0P) by about 20.16% and 14.5% at 7 and 28 days, correspondingly. Likewise, the mix’s strengths with 0.5% PPF only (M0D0.5P) were inferior to that of mix M0D0P at 7 and 28 days by 7.5% and 5.33%, respectively. The decline in compressive strength with DPF addition was mainly ascribed to the high wettability and hydrophilicity of the DPF, which increases air entrainment in the fresh concrete and, when hardened, upsurges the pores in the cement matrix, consequently causing premature failure and reduced strength. Another reason is that the lack of proper bonding between the matrix and DPF creates a vulnerable interfacial transition zone within the matrix, forming a weak path for premature failure [11,64]. Findings by Boumhaout, Boukhattem [65], and Adamu, Ibrahim [66] have all shown that the compressive strengths of concrete and mortar are reduced with DPF addition. The drop in strength of the concrete with the addition of PPF was linked to the loss of homogeneity of the fresh concrete and the escalation of voids and pores in the cementitious matrix due to the fiber effect and the formation of weak interfacial bonds between the cement matrix and the fibers [21,67]. Many studies have also reported a reduction in compressive strength of cement composites with the addition of PPF [7,21,67]. The decline in compressive strength was less with the addition of PPF than with DPF. The DPF was less compatible and caused more severe effects in concrete because it is a natural fiber with less tensile strength and elasticity than PPF.

Furthermore, the DPF contains higher hydrophilicity and hemicellulose content; this makes it absorb more water and entraps more air during mixing, thereby causing more pores within the hardened cement matrix than the PPF, thus aiding more in a reduction in compressive strength. The addition of hybrid DPF and PPF to concrete also caused a decline in the compressive strength of concrete. Thus, the strength reduction was less severe than when only DPF was added, and it was more pronounced than when only PPF was added. This could be described by relating the compressive strength values of mix M0.25D0.25P with the mix containing 0.5% DPF (M0.5D0P) and the mix containing 0.5% PPF (M0D0.5P) in Figure 6. Mix M02.5D0.25P has higher strengths at 7 and 28 days compared to M0.5D0P, but has lower strengths compared to M0D0.5P. Similarly, hybrid mixes with higher DPF and lower PPF exhibit lower compressive strength than mixes with lower DPF and higher PPF. By relating the compressive strengths of mixes M1D0.5P and M0.5D1P, the former demonstrated lower strengths than those at both 7 and 28 days. This effect was due to the higher adverse effects of DPF compared to PPF in the concrete. Hence, hybrid DPF-PPF mixes can be produced with lesser DPF and higher PPF for higher strengths, where partially or fully replacing the DPF with PPF can lower the concrete’s strength loss.

The mechanical performance of fiber-reinforced concrete is significantly influenced by the ITZ between the fiber and cement matrix. In this study, both date palm fiber (DPF) and polypropylene fiber (PPF) exhibited relatively weak bonding with the cement matrix, as evidenced by SEM analysis. This poor adhesion can be attributed to the hydrophilic nature of DPF, which leads to insufficient chemical compatibility with the cement paste, and the hydrophobic surface of PPF, which limits wetting and mechanical anchorage. The presence of fibers also introduces additional porosity due to increased air entrapment during mixing and water absorption by natural fibers like DPF. This contributes to a weakened ITZ between the cement paste and the fibers. To mitigate these effects and enhance the mechanical properties, several strategies could be considered. These include (1) surface modification of fibers, such as alkali treatment for DPF or plasma treatment for PPF, to improve interfacial bonding, (2) use of supplementary cementitious materials (SCMs) like silica fume or nano silica to densify the matrix and reduce porosity and (3) improved dispersion techniques including the use of sonication, to minimize fiber agglomeration and void formation.

### 3.4. Splitting Tensile Strength

**Figure 7:**
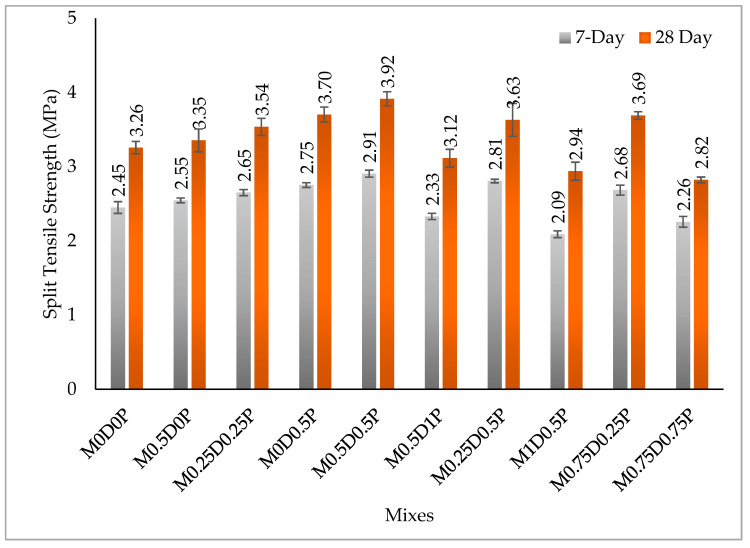
Results of splitting tensile strength.

Figure 7 displays the splitting tensile strengths of the hybrid DPF-PPF-reinforced concrete mixes, where it increases with the curing duration because of ongoing hydration processes. The addition of up to 1% dosage of DPF or PPF or a hybrid of DPF and PPF resulted in an improvement in the tensile strength of the concrete. This is evident by comparing the different concrete mixes with the control mix. With respect to the concrete mix with only DPF, the tensile strength of mix M0.5D0P was marginally greater than the control mix (M0D0P) at 7 and 28 days by about 3.94% and 3.0%, respectively. For the concrete mix containing only PPF, the tensile strengths of M0D0.5P were greater at 7 and 28 days by 12.39% and 13.68%, respectively, compared to mix M0D0P.

Additionally, for the hybrid mixes, the strengths of mix M0.25D0.25P were higher at 7 and 28 days by 8.23% and 8.6%, while for mix M0.25D0.5P, it was higher at 7 and 28 days by 14.6% and 11.43%, respectively, relative to the reference mix. The increment in tensile strength with incorporation of both DPF and PPF can be credited to the fiber effects that occur in the concrete matrix, which includes the energy transfer system and the ability to bridge the cracks formed due to load applications. This resulted in a significant rise in the post-failure load resistance and higher tensile strength. The fibers enhanced the concrete’s resistance to initiation of cracks, leading to increased tensile strength [4,11,54]. Previous findings by Ibrahim, Adamu [11], and Althoey, Hakeem [68] have also reported enhancement in the splitting tensile strength of cement composite with the addition of DPF. Furthermore, the outcomes of this study were consistent with earlier findings [64,68,69] where they reported enhancement in the tensile strengths of cementitious composites with the addition of PPF.

From Figure 7, in the concrete mixes’ series, the mixes containing only PPF exhibit the highest tensile strength compared to those containing a hybrid of DPF and PPF. Meanwhile, mixes containing only DPF have the lowest tensile strengths. For instance, by comparing the three-mix series containing an equal proportion of the fibers (0.5%) to the control (M0D0P), mix M0D0.5P has a tensile strength value that is higher at 7 and 28 days by 12.39% and 13.68%, respectively, and mix M0.25D0.25P has tensile strengths values higher at 7 and 28 days by 8.23% and 8.6%, respectively. Mix M0.5D0P’s tensile strengths were higher by 3.94% and 3.0% at 7 and 28 days, respectively, compared to the control. In addition, the hybrid DPF-PPF mixes containing more PPF and less DPF contents have higher strengths than the hybrid fiber mixes containing more DPF and less PPF dosages. The tensile strength of mix M0.5D1P was 2.33 MPa and 3.12 MPa at 7 and 28 days, while M1D0.5P had a tensile strength value of 2.09 MPa and 2.94 MPa at 7 and 28 days. Therefore, from the findings in this research, PPF was more effective in improving the tensile strength of concrete than DPF.

Furthermore, using hybrids of DPF and PPF was better in terms of enhancement in the tensile strength than using DPF only. This occurs because the PPF has higher tensile strength and elastic modulus than the DPF. Furthermore, as the DPF is a natural fiber (vegetative fiber), its high hydrophilicity, wettability, and hemicellulose reduce its compatibility and adhesion with the cement paste, limiting the progress of the tensile strength of the concrete. The outcomes in this study were consistent with those of Acosta-Calderon, Gordillo-Silva [62], who used a hybrid of PPF and sisal fibers, and they reported PPF to be more effective in improving the tensile strengths of concrete compared to sisal fiber, which is a natural fiber.

There was a decline in the tensile strength of the concrete when a higher dosage (1.5%) of the hybrid DPF and PPF was added. This can be seen by comparing the tensile strengths of mixes M1D0.5P, M0.5D1P, and M0.75D0.75P with that of the control mix, where all the aforementioned mixes have lower tensile strengths in comparison to M0D0P. This might be caused by increased pore volume in the concrete matrix when higher dosages of DPF and PPF were added, resulting in premature failure and reduced tensile strength. Another factor can be linked to the poor adhesion between the fibers, especially the DPF and cement matrix, which creates weak interfacial paths for premature failure. These findings were inconsistent with previous studies, where Yuan and Jia [21] reported reductions in the tensile strength of concrete when more than 1% PPF was added and Hosseinzadeh, Salehi [3] also reported similar observations. Ozerkan, Ahsan [70] also reported a reduction in tensile strength with the addition of more than 1% DPF to mortar.

### 3.5. Flexural Strength

**Figure 8:**
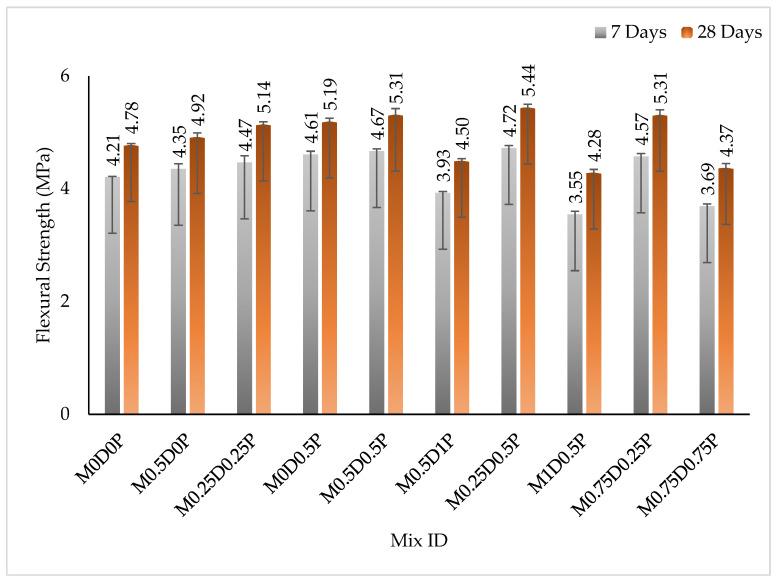
Results of flexural strength.

The results of the flexural strengths of the concrete containing different hybrid proportions of DPF and PPF are shown in Figure 8. The flexural strengths of the concrete improved with the age of curing, ascribing it to the progression of the hydration reaction with time. Adding single or hybrid DPF and PPF enhanced the concrete’s flexural strength (bending resistance). The flexural strength of the mix with only 0.5% DPF, i.e., M0.5D0P, was higher than for mix M0D0P at 7 and 28 days by 3.41% and 2.97%, respectively. Similarly, the flexural strength for the mix with 0.5% PPF only, i.e., M0D0.5P, was higher than that of mix M0D0P by 9.48% and 8.73% at 7 and 28 days, respectively. For the mixes with hybrid DPF and PPF, the flexural strength of mix M0.25D0.25P was higher than mix M0D0P at 7 and 28 days by 6.14% and 7.65%, respectively. The enhancement in the bending resistance of concrete with the addition of fibers can be ascribed to the bridging role of fibers, which impedes crack elongation and mitigates crack widths. The crack bridging effect also increases the resistance to applied loads after cracking, thereby improving the flexural performance of the concrete [16,67]. The results of flexural strengths show that the PPF contributed more to improving the concrete’s flexural performance than the PPF. The latter has higher flexural strengths when compared to the mixes containing equal proportions of single fibers only, i.e., M0.5D0P and M0D0.5P.

Additionally, concerning the concrete mixes containing hybrid fibers, comparing two mixes with the exact dosage of the hybrid fibers, the mixes with higher PPF content demonstrated superior flexural strengths compared to mixes with higher DPF. As a comparison, the mix M0.5D0.P has higher strengths than the mix M0.75D0P. Similarly, mix M0.5D1P has greater flexural strength than mix M1D0.5P. This superior improvement in flexural strength by the PPF about DPF is due to its higher tensile strength and elastic modulus, which are better than those of DPF. This gives the PPF higher ductile behavior, better ability to effectively bridge cracks in the cement matrix, and more substantial enhancement in the flexural strength compared to DPF. These findings concur with those of Acosta-Calderon and Gordillo-Silva [62], who observed more significant flexural strength improvements in concrete using PPF than in sisal fibers, a natural fiber. As shown in Figure 8, when higher dosages of the hybrid DPF and PPF above 1% were added, a reduction in the flexural strength of the concrete was observed. This reduction is more pronounced in the mixes with higher DPF and lower PPF. For instance, the flexural strength of mixes M1D0.5P, M0.5D1P, and M0.75D0.75P were lower by 10.27%, 5.85%, and 8.49%, respectively, at 28 days compared to mix M0D0P. The decline in flexural strength with the incorporation of a high content of hybrid fibers can be associated with the following factors: (i) escalation of pore volume, which creates a fragile path for premature failure to occur; (ii) non-compatibility between the DPF and PPF due to variations in their properties causes them to purely bond with the cement paste [11,64]. These findings, with results reported by Yuan and Jia [21], show an increase in flexural strength of concrete with the addition of PPF greater than 1%. Ozerkan, Ahsan [70] also observed a decrease in the flexural strength of concrete with the incorporation of more than 1% DPF.

#### Flexural Toughness and Ductility

**Figure 9:**
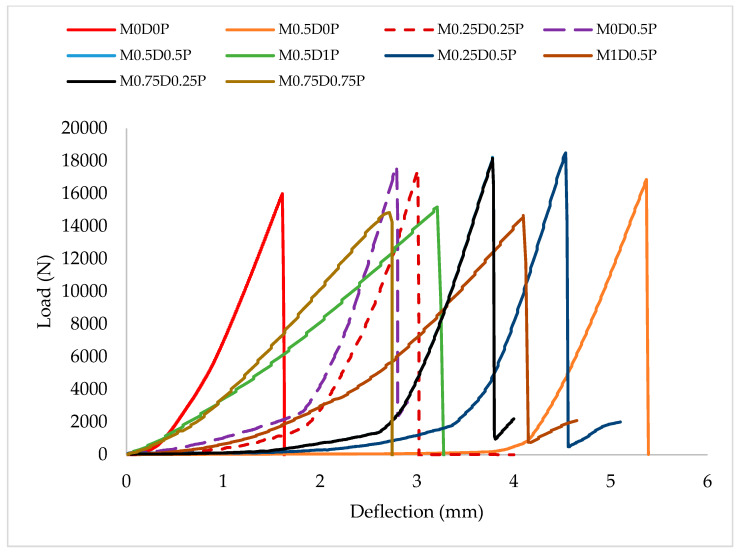
Load–deflection curve for the hybrid DPF-PPF concrete mixes.

One of the benefits of incorporating fiber into concrete is to reduce its brittle nature and enhance its toughness. The concrete’s toughness (energy absorption capacity) is the area under the load–deflection curve under flexural loads. The first cracking, ultimate load, and the structural elements’ stiffness significantly influenced the load–displacement curve’s behavior [15,68]. The load–deflection plots for the hybrid DPF-PPF-reinforced concrete are depicted in Figure 9. The load–deflection curve for the control mix (M0D0P), which does not contain any fiber, exhibits a steep initial slope, and its area under the load–deflection curve is narrow, indicating a high stiffness and low toughness (energy absorption capacity). Furthermore, it fails suddenly after reaching the ultimate load, demonstrating negligible post-cracking deformation and brittle behavior. All the mixes containing different proportions of DPF and PPF, such as M0.25D0.25P, M0.5D0.5P, M0.75D0.25P, and other similar mixes, exhibit a gentler initial slope, more extensive area under their curves, and a gradual decline or plateau, demonstrating that the addition of fiber reduced the brittle nature, improving the ductile and post-cracking behavior of the concrete. The mixes containing higher content of the hybrid fibers such as M1D0.5P, M0.5D1P, and M0.75D0.75P exhibited the gentlest initial slopes and the most expansive areas under their load–deflection curves, even though they had the lowest ultimate loads. This explains that these mixes have the highest ductility, energy absorption capacity, and post-cracking behavior compared to the other mixes. This behavior suggests that even though the high content of fibers in the concrete reduced its ultimate load resistance, the addition of a high dosage of the fiber significantly enhanced the concrete’s energy absorption and toughness by mitigating brittle fracture and promoting crack bridging.

**Figure 10:**
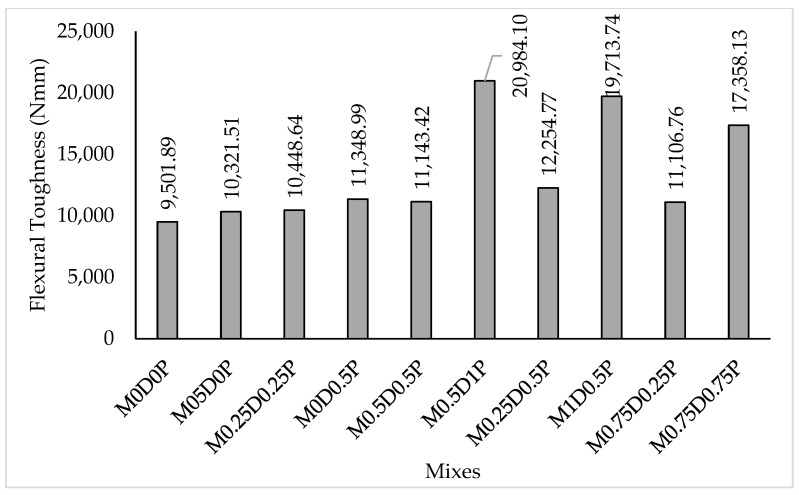
Flexural toughness for the hybrid DPP-PPF concrete mixes.

The energy absorption capacity for all the mixes was computed by calculating the areas under their load–deflection curves, as shown in Figure 10, which justified the improvements in ductility and flexural toughness of the concrete with the incorporation of DPF and PPF, where all the mixes containing either DPF, PPF or hybrid DPF and PPF have higher flexural toughness compared to the control mix (M0D0P). Another absorption was that the PPF is more effective in improving the concrete’s ductile behavior and energy absorption than DPF. This can be justified by comparing the toughness of the mix series with 1.5% hybrid fibers, where M0.5D1P has the highest toughness, followed by M0.75D0.75P, while M0.5D1P has the lowest toughness in the series. Similarly, for the mix series with 0.5% fiber, M0.5D0P has lower flexural toughness than M0D0.5P. This superiority of the PPF over DPF is ascribed to the higher tensile strength, modulus of elasticity, and elongation at break compared to DPF; this gives it a more crack-bridging effect.

### 3.6. Water Absorption

**Figure 11:**
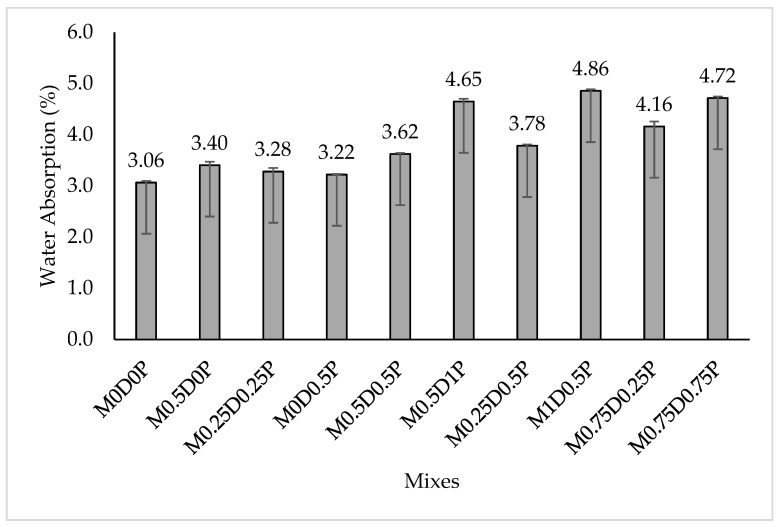
Water absorption results.

The water absorption test is used as a measure of the durability performance of the hybrid DPF-PPF concrete, and the result is depicted in Figure 11. The water absorption of concrete increases with an increase in percentage addition of either the single fibers (DPF or PPF) or hybrid DPF-PPF. The water absorption of mixes M0.5D0P, M0D0.5P, and M0.25D0.25P was higher by 11%, 5.06%, and 7.01%, respectively, compared to M0D0P. The surge in water absorption with the addition of DPF and PPF can be ascribed to the creation of a weak interfacial bond between the cement paste, aggregates, and the fiber, leading to the formation of a more extensive pore network in the concrete, escalating the water absorption [68]. Considering each fiber, the DPF, which is a natural/vegetable fiber, contains a substantial amount of cellulose, hemicellulose, and lignin, which gives it high hydrophilicity, high wettability and the ability to absorb more water during mixing or curing, reaching up to 300% of its dry weight. After hardening, the water absorbed by the DPF during mixing dries up and generates micropores within the cementitious matrix, thus increasing water absorption.

Additionally, the water absorbed by the DPF during curing makes it swell and builds up microcracks, which escalates the rate of water absorption [12,13,71]. For the PPF, as the macro-fiber type was used, it affects the workability, and its dispersion within the concrete can be challenging; improper distribution of the PPF can increase porosity within the cement paste, thus increasing water absorption [69]. A previous study by Fallah and Nematzadeh [72] also reported an increased water absorption in concrete with the addition of 0.4% or higher dosage of PPF. Althoey, Hakeem [68] also reported an escalation of water absorption with up to 0.6% PPF. Adding DPF as a single fiber or hybrid with the PPF causes more increment in water absorption. For the single-fiber-reinforced concretes, comparing their water absorption values with the control mix, mix M0.5D0P has the highest increment of 11% while M0D0.5P has an increment of 5.06%. For the hybrid fiber-reinforced concretes, the control mix, the water absorption of mixes M1D0.5P, M0.5D1P, and M0.75D0.75P was increased by 58.43%, 51.62%, and 53.9%, respectively. The higher increment in water caused by the DPF was linked to its additional hydrophilicity due to its cellulose, hemicellulose, and lignin content, giving it a greater absorption ability than the PPF. The PPF is somewhat hydrophobic, and it cannot absorb water. Therefore, it does not affect water absorption hydrophilically.

### 3.7. FESEM Analysis

**Figure 12:**
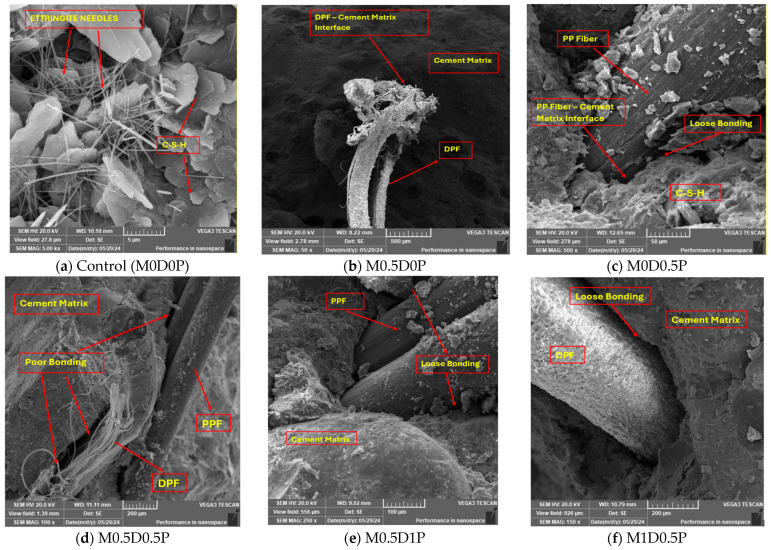
Microstructural morphology results.

The scanning electron microscopy (SEM) images of representative mixes are presented in Figure 12. The SEM image of the control mix (M0D0P) clearly shows the presence of hexagonal crystalline hydration products, which consist of calcium silicate hydrates. Additionally, needle-shaped prismatic crystals of ettringite and a reticular network crystalline structure of calcium hydroxide are prominently observed within the pores of the cement matrix. These observations signify several important characteristics of the control mix. Firstly, hexagonal crystalline hydration products indicate a high-quality cementitious matrix. The formation of these crystalline structures suggests a well-developed hydration process, contributing to the overall strength and durability of the concrete. Secondly, the presence of needle-shaped prismatic ettringite crystals and the reticular network crystalline structure of calcium hydroxide further confirms the dense and compact nature of the control mix. The well-defined and interconnected crystal formations within the cement matrix indicate a high degree of compaction during the mixing and curing. These microstructural findings align with the highest compressive strength value of the control mix. The dense structure and compaction observed within the cement matrix contribute to improved load transfer mechanisms and enhanced resistance to crack propagation, resulting in higher compressive strength values. Figure 12b shows the morphology of mix M05D0P, and it shows that the DPF does not have strong and tight bonding with the cement matrix, meaning poor adhesion exists, which might be due to the hydrophilic nature of the DPF due to its high hemicellulose and lignin content. This causes a reduction in the strength and an increase in the porosity of the concrete [12]. Figure 12c presents the morphology of mix M0D0.5P. The image depicts a noticeable lack of strong adhesion between the cement paste and PPF, indicating a detrimental effect on the concrete’s strength properties. The hydrophobic nature of the PPF, a synthetic fiber, can cause this.

The hydrophobic characteristic impedes the contact between the PPF and the cement matrix, resulting in a less resilient bond that can potentially diminish the concrete’s overall strength [73]. Compared to the mix containing only DPF (M0.5D0P, Figure 12b), the PPF demonstrated tighter adhesion and bonding with the cement matrix, with a more densified microstructure. PPF is not hydrophilic; no chemical compounds like hemicellulose or lignin will reduce its adhesion to the cement paste. The hydrophilicity of the DPF negatively affects the adhesion of the fibers compared to the hydrophobicity of the PPF. This can be further explained by studying the morphology of mix M0.5D0.5P (Figure 12d), where the poor bonding between the DPF and cement paste creates a more significant transition path than the interfacial layer between PPF and cement paste. Figure 12e and Figure 12f display mixes M0.5D1P and M1D0.5P, respectively, which involve the combination of DPF and PPF at high dosages of 1.5% each. The micromorphology images reveal less dense matrices than other mixes, implying reduced compaction and increased porosity.

Additionally, the bonding zone between the fiber types and the cement matrix appears loosely connected, indicating inadequate bonding between the fibers and the matrix. This loose bonding zone and insufficient dispersion of the fibers contribute to the relatively low compressive strength results observed in mixes M0.5D1P and M1D0.5P. The high fiber dosages in these mixes impede compaction and lead to a less favorable interphase between the fibers and the matrix. The morphology of M0.5D1P (Figure 12e) tends to be denser with lesser poor bonding than that of M1D0.5P (Figure 12f) due to the substantial adverse effect of the DPF compared to PPF.

**Figure 13:**
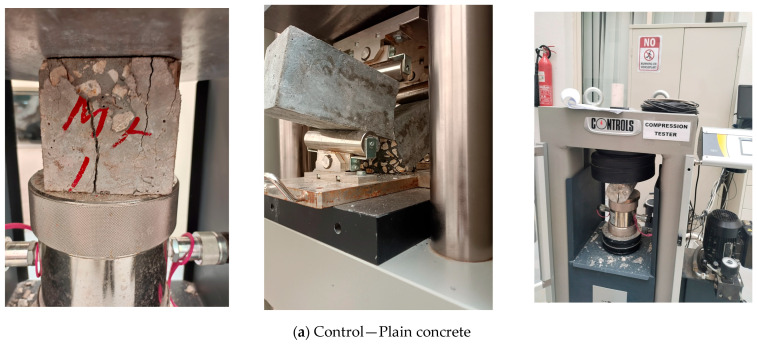

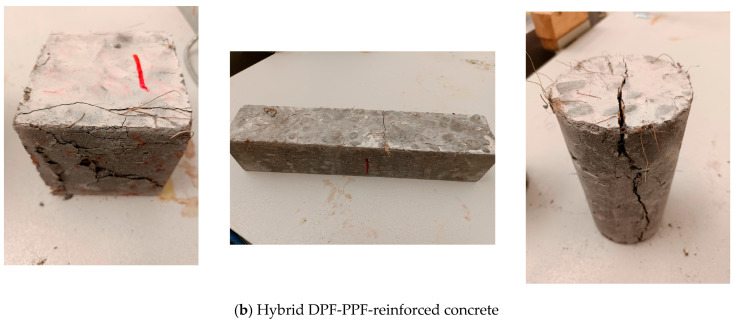
Destroyed samples after testing.

Figure 13 shows the failure (destruction) of the control (plain) concrete and hybrid DPF-PPF concrete samples. The plain concrete samples exhibit catastrophic failures with no bridging effect under compression, flexural or tensile loads (Figure 13a), where the samples breaks with larger cracks after failing. However, for the hybrid fiber-reinforced concrete samples (Figure 13b), crack bridging effects can be observed, where the samples still remain intact without breaking after failing under compression, flexural and tensile loads. This is due to the fiber effects in the concrete mixes.

### 3.8. Mercury Intrusion Porosimetry (MIP)

**Figure 14:**
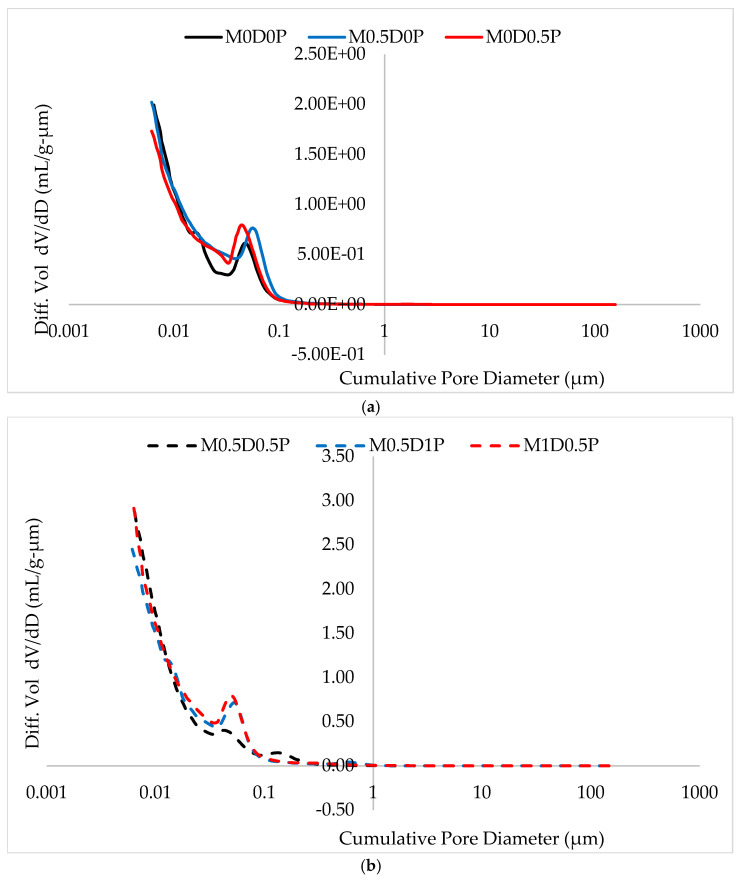
(**a**) Mixes’ pore size distribution I. (**b**) Mixes’ pore size distribution II.

**Figure 15:**
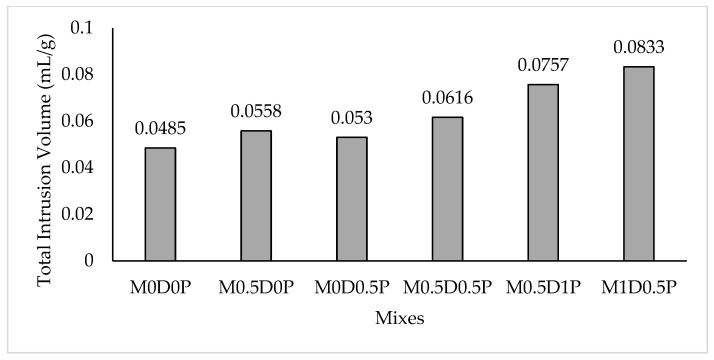
Total intrusion for all mixes.

As a comparative analysis, Figure 14a,b present the pore size distribution of fiber-reinforced matrices about the control matrix (without fiber) based on their respective differential volumes. Up to 6 nm (the lower limit of measurement using MIP), all matrices exhibited a nearly unimodal pore size distribution, characterized by a peak pore diameter of approximately 10 nm, followed by a sharp decline in pore sizes that was more significant than the value for the control mix (which exhibited one of the highest compressive strength values among all the studied mixes). A slight variation was observed among the fiber-containing mixes, where a similar sharp decrease in pore diameter was observed, except for a small peak in the range of 0.05 μm for mixes M0.5D0.5P, M0.5D1P, and M1D0.5P, as shown in Figure 14b, and 0.06 μm for mixes M0.5D0P and M0D0.5P, as depicted in Figure 14a. No pores larger than 100 nm were found. Pores larger than 80 μm were attributed to micro-cracks created during sample cutting for compatibility with the MIP device. These results were further corroborated by the total intrusion results presented in Figure 14, where the mixes with higher DPF and PPF contents, such as M0.5D0.5P, M0.5D1P, and M1D0.5P, exhibit higher total intrusion volume relative to mix M0D0P and other mixes with lower fiber contents. This is due to the surge in porosity caused by the fiber in the cement matrix. The mixes with higher DPF content demonstrated a higher pore volume than those with higher PPF. For example, mix M1D0.5P has a higher total intrusion volume than mix M0.5D1P, as shown in Figure 15. This is due to DPF being a plant-based fiber containing higher hydrophilicity and hemicellulose content, which means that it absorbs more water and entraps more air during mixing, thereby causing more porosity in the hardened cement matrix than the PPF [11,12].

**Figure 16:**
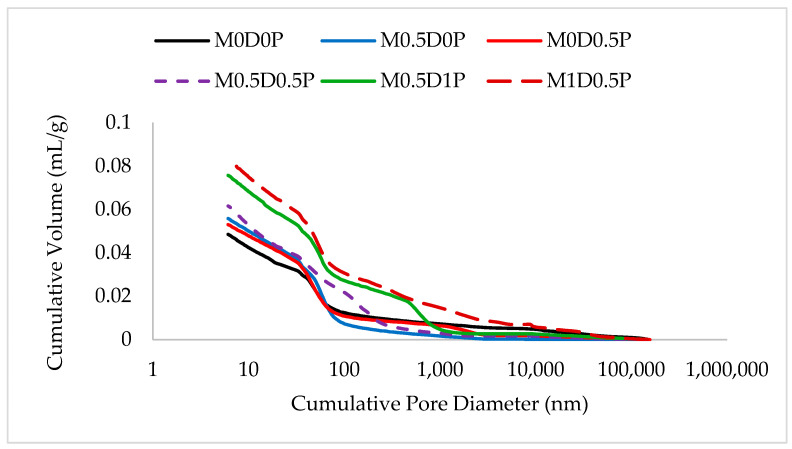
Cumulative volume vs. pore diameter for all mixes.

Figure 16 illustrates the cumulative pore volume (CPV) of the fiber-reinforced mixes compared to the control mix (without fiber). The figure demonstrates that the CPV increases with the addition of fibers, particularly in mixes M0.5D1P and M1D0.5P, regardless of the fiber type. The inclusion of DPF content leads to a slight increase in pore values, with the highest pore values observed in mix M1D0.5P, which contained 1% DPF and 0.5 PPF and an overall fiber content of 1.5%, particularly in pores with diameters larger than 30 nm. Different fiber types and modes of fiber addition exhibit varying effects on the CPV. A comparison of the curves of mixes M0.5D0P, M0D0.5P, and M0.5D0.5P reveals that the CPV of concrete with a matrix strength equivalent to that of mix M0D0.5P, followed by M0.5D0P exhibited the lowest cumulative pore volume, indicating achieving high compaction levels with adding 0.5% DPF or PPF. In conclusion, the cumulative pore values are directly related to the compressive strength, as evidenced by the similar values observed for the control mix, M0.5D0P, M0D0.5P, and M0.5D0.5P, and the increased values for mixes M0.5D1P and M1D0.5P, which exhibited lower compressive strength values and higher fiber contents, suggesting a higher likelihood of fiber agglomeration due to their larger volume within the matrices.

MIP is a widely accepted approach for assessing the porosity and pore size distribution of porous materials, including cement-based materials. Table 4 summarizes the results of the pore size distribution and porosity of cement matrices with and without fiber addition at 28 days. Furthermore, pores in cement-based materials are categorized into four types: harmless pores (pore size smaller than 20 nm), minor harmful pores (pore size between 20 nm and 100 nm), harmful pores (pore size between 100 nm and 200 nm), and serious harmful pores (pore size larger than 200 nm). From Table 4, it can be observed that compared to the control mix (M0D0P), the mixes with the highest fiber reinforcement content (1.5%)—M0.51DP and M1D0.5P—experienced an increase in both the total pore volume and total porosity at 28 days, reaching 14% and 16%, respectively, compared to 11% for the control mix. Similarly, the total intrusion volume of pores reached approximately 0.08 mL/g for M0.51DP and M1D0.5P, compared to 0.06 mL/g for the control mix.

In contrast, mixes M0.5D0P and M0D0.5P exhibited a total porosity of approximately 12% and a total intrusion volume of approximately 0.05 mL/g, indicating an optimal level of compaction for the fiber-incorporated mixes. This trend is further supported by comparing bulk density values, with the control mix exhibiting the highest bulk density. In contrast, mix M0.51DP and M1D0.5P demonstrated the lowest bulk densities due to the effects of high dosage of fiber addition.

### 3.9. Discussion

The experimental investigation conducted in this study provides valuable insights into the mechanical behavior and durability performance of concrete reinforced with hybrid date palm fiber (DPF) and polypropylene polymer fiber (PPF). The incorporation of both natural and synthetic fibers significantly influences various properties of concrete, including workability, density, compressive strength, tensile strength, flexural toughness, ductility, and water absorption characteristics. The observed reduction in workability and fresh density upon fiber addition is primarily attributed to the hydrophilic nature of DPF and its ability to absorb significant amounts of mixing water, thereby reducing the effective water-to-cement ratio. In contrast, PPF, being hydrophobic, does not absorb water but may interfere with the flowability of the mix due to its fibrous structure. The decline in compressive strength with increasing fiber content can be linked to poor fiber–matrix adhesion and increased porosity, particularly in mixes containing higher proportions of DPF. However, the inclusion of PPF, with its superior tensile strength and better compatibility with the cement matrix, mitigates some of these adverse effects, especially in hybrid combinations where PPF partially replaces DPF. In terms of tensile and flexural performance, both DPF and PPF contribute positively by bridging cracks and enhancing post-cracking resistance. PPF demonstrates superior improvements compared to DPF due to its higher tensile strength and elongation at break, enabling more effective crack control and energy dissipation. Hybridization of the two fibers further enhances the toughness and ductility of the concrete, particularly when the dosage remains below 1%. These findings suggest that a balanced combination of DPF and PPF can optimize the mechanical performance of concrete without compromising its structural integrity excessively. Durability assessments reveal that fiber addition increases water absorption and pore volume, with DPF having a more pronounced effect due to its high hemicellulose and lignin content, which enhances hydrophilicity and microporosity. While this poses potential concerns regarding long-term durability, the use of PPF in hybrid combinations can help reduce the negative impact by improving the overall compactness of the matrix and minimizing capillary action. Microstructural analyses using SEM and MIP confirm the formation of weak interfacial transition zones around DPF due to poor bonding with the cement matrix, leading to increased porosity and reduced strength. Conversely, PPF exhibits relatively better integration within the matrix, although full compatibility remains a challenge. The presence of larger pores in mixes with high fiber dosages (>1%) further supports the observation of declining mechanical performance at excessive fiber contents, likely due to agglomeration and improper dispersion. From a sustainability and cost-effectiveness perspective, the hybrid use of DPF and PPF offers a promising alternative for enhancing the performance of concrete while leveraging the benefits of locally available natural fibers and industrially produced synthetic fibers. The optimal balance between mechanical enhancement and durability preservation appears to occur with controlled hybrid proportions, particularly those favoring PPF over DPF.

## 4. Conclusions

This research examined the impacts of hybridization of natural (DPF) and synthetic fiber (PPF) on concrete’s mechanical and durability performance. The key findings can be summarized as follows:

1. Adding DPF and PPF reduced the workability and fresh density of concrete, with DPF exhibiting a more significant reduction due to its higher hydrophilicity and hemicellulose content.
2. The compressive strength of concrete is reduced by adding either single DPF or PPF or their hybrid to the concrete. Furthermore, DPF exhibits a more detrimental effect on the strength of concrete due to its poor compatibility with the cement matrix and high hydrophilicity. On the other hand, the concrete’s tensile strengths (flexural and split tensile strengths) improved with the addition of either single fiber (DPF or PPF) or their hybrid. These improvements were limited to when the dosage of the single fiber or hybrid fibers did not exceed 1%. PPF demonstrated superior improvements in hybrid fiber-reinforced concrete and single-fiber-reinforced concrete compared to DPF due to its higher tensile strengths and elasticity.
3. Adding either DPF or PPF or their hybrid enhanced the energy absorption capacity, toughness, and ductility of the concrete, which increases with an increment in fiber addition. Therefore, adding either a single DPF or PPF or hybridization of DPF and PPF into the concrete promotes post-cracking behavior and inhibits crack propagation, which means they can be used in plain or reinforced concrete. Comparing the two fibers, PPF demonstrated higher improvement due to its superior elasticity and tensile strength compared to DPF.
4. The durability of the concrete measured in terms of water absorption and mercury intrusion porosimetry showed that the fibers increased the water absorption and escalated the pore volume in the concrete matrix. The DPF, owing to its high hemicellulose and hydrophilicity, caused a higher increment in the porosity and absorption of the concrete, raising potential durability concerns.
5. The best performance was observed in hybrid mixes with controlled proportions of PPF and DPF, balancing mechanical properties and durability. Excessive fiber content (>1.5%) reduced strength due to fiber agglomeration and increased porosity.
6. The microstructural morphology showed that both DPF and PPF were poorly bonded with the cement matrix, resulting in premature failure and reduced compressive strength. The DPF exhibits the worst bonding with the cement matrix due to its high hydrophilicity, aiding in a more pronounced reduction in strength and increased porosity.
7. To reduce the water absorption of DPF, it is recommended to explore fiber treatment methods such as alkali treatment, silane treatment, or acetylation to decrease hydrophilicity. Applying hydrophobic coatings or sizing (e.g., epoxy resins, nano-silica suspensions) may also serve as an effective moisture barrier. Additionally, optimizing fiber dosage and combining DPF with hydrophobic synthetic fibers like polypropylene (PPF) could help balance water absorption while maintaining mechanical performance.

## Figures and Tables

**Table 1:** Properties of cement used.

Oxides/Property	Composition/Value
SiO_2_ (%)	19.71
Al_2_O_3_ (%)	5.39
Fe_2_O_3_ (%)	3.40
CaO (%)	65.18
SO_3_ (%)	3.51
K_2_O (%)	1.22
MgO (%)	0.91
TiO_2_ (%)	0.24
Na_2_O (%)	0.17
MnO (%)	0.18
P_2_O_5_ (%)	0.09
Specific gravity	3.15
Bulk density (kg/m^3^)	1440

**Table 2:** Properties of polypropylene polymer fiber and date palm fiber.

Properties	Values	
	PPF ^1^	DPF ^2^
Appearance/Color	Colorless	Light to dark brown
Apparent density (kg/m^3^)	910	877.43 ± 4.8
Diameter (mm)	1.0 ± 5%	0.2–1.0
Fiber length (mm)	56 ± 5%	20–30
Aspect ratio	56 ± 5%	30–100
Tensile strength (MPa)	500 ± 7.5%	203.24 ± 30
Elastic modulus (GPa)	10.0 ± 10%	3.35 ± 1.5
Elongation at break (%)	21 ± 1.5	13.5 ± 2
Melting point (°C)	160–165	−

^1^ PPF = polypropylene polymer fiber, ^2^ DPF = date palm fiber.

**Table 3:** Mix proportions and constituent materials.

Mix ID	Variables (%)		Quantities of Materials in kg/m^3^						
	DPF	PPF	Cement	Fine Agg	Coarse Agg	DPF	PPF	Water	S.P
M0D0P (Control)	0	0	420	755	975	0.00	0.00	157.25	4.20
M0.5D0P	0.5	0	420	755	975	2.10	0.00	157.25	4.20
M0.25D0.25P	0.25	0.25	420	755	975	1.05	1.05	157.25	4.20
M0D0.5P	0	0.5	420	755	975	0.00	2.10	157.25	4.20
M0.5D0.5P	0.5	0.5	420	755	975	2.10	2.10	157.25	4.20
M0.5D1P	0.5	1	420	755	975	2.10	4.20	157.25	4.20
M0.25D0.5P	0.25	0.5	420	755	975	1.05	2.10	157.25	4.20
M1D0.5P	1	0.5	420	755	975	4.20	2.10	157.25	4.20
M0.75D0.25P	0.75	0.25	420	755	975	3.15	1.05	157.25	4.20
M0.75D0.75P	0.75	0.75	420	755	975	3.15	3.15	157.25	4.20

PPF = polypropylene polymer fiber, DPF = date palm fiber and S.P = superplasticizer.

**Table 4:** Summary of intrusion data.

Factor/Mix	M0D0P (Control)	M0.5D0P	M0D0.5P	M0.5D0.5P	M0.5D1P	M1D0.5P
Total Intrusion Volume	0.0485 mL/g	0.0558 mL/g	0.0530 mL/g	0.0616 mL/g	0.0757 mL/g	0.0833 mL/g
Total Pore Area	7.604 sq-m/g	8.621 sq-m/g	7.809 sq-m/g	10.183 sq-m/g	10.122 sq-m/g	10.998 sq-m/g
Median Pore Diameter (Volume)	0.0464 μm	0.0476 μm	0.0441 μm	0.0491 μm	0.0554 μm	0.0550 μm
Median Pore Diameter (Area)	0.0114 μm	0.0137 μm	0.0140 μm	0.0105 μm	0.0122 μm	0.0119 μm
Average Pore Diameter (4 v/A)	0.0255 μm	0.0259 μm	0.0271 μm	0.0242 μm	0.0299 μm	0.0303 μm
Bulk Density	2.3112 g/mL	2.2149 g/mL	2.2837 g/mL	2.3081 g/mL	2.1994 g/mL	2.1005 g/mL
Apparent (Skeletal) Density	2.6028 g/mL	2.5270 g/mL	2.5978 g/mL	2.6906 g/mL	2.6388 g/mL	2.5460 g/mL
Porosity	11.20%	12.35%	12.09%	14.22%	16.65%	17.50%

## Data Availability

Data are contained within the article.

## References

[1] Fuzail Hashmi A., Shariq M., Baqi A. (2020). Successive sustained loading effect on the long-term deflection of flat slab. SN Appl. Sci..

[2] Shah S.P., Swartz S.E., Ouyang C. (1995). Fracture Mechanics of Concrete: Applications of Fracture Mechanics to Concrete, Rock and Other Quasi-Brittle Materials.

[3] Hosseinzadeh H., Salehi A.M., Mehraein M., Asadollahfardi G. (2023). The effects of steel, polypropylene, and high-performance macro polypropylene fibers on mechanical properties and durability of high-strength concrete. Constr. Build. Mater..

[4] Hou L., Wen B., Huang W., Zhang X., Zhang X. (2023). Mechanical Properties and Microstructure of Polypropylene–Glass-Fiber-Reinforced Desert Sand Concrete. Polymers.

[5] Li F., Jin S., Cheng P., Wang Z., Yang Z. (2024). Study on Mechanical Properties and Carbon Emission Analysis of Polypropylene Fiber-Reinforced Brick Aggregate Concrete. Polymers.

[6] Del Savio A.A., La Torre Esquivel D., García Landeo J.M. (2023). Post-Cracking Properties of Concrete Reinforced with Polypropylene Fibers through the Barcelona Test. Polymers.

[7] Yaqin C., Haq S.U., Iqbal S., Khan I., Room S., Khan S.A. (2024). Performance evaluation of indented macro synthetic polypropylene fibers in high strength self-compacting concrete (SCC). Sci. Rep..

[8] Balea A., Fuente E., Monte M.C., Blanco Á., Negro C. (2021). Fiber reinforced cement based composites. Fiber Reinforced Composites.

[9] Bentur A., Mindess S. (2006). Fibre Reinforced Cementitious Composites.

[10] Al-Oqla F.M., Alothman O.Y., Jawaid M., Sapuan S., Es-Saheb M. (2014). Processing and properties of date palm fibers and its composites. Biomass and Bioenergy: Processing and Properties.

[11] Ibrahim Y.E., Adamu M., Marouf M.L., Ahmed O.S., Drmosh Q., Malik M.A. (2022). Mechanical performance of date-palm-fiber-reinforced concrete containing silica fume. Buildings.

[12] Adamu M., Alanazi F., Ibrahim Y.E., Alanazi H., Khed V.C. (2022). A comprehensive review on sustainable natural fiber in cementitious composites: The date palm fiber case. Sustainability.

[13] Benaniba S., Driss Z., Djendel M., Raouache E., Boubaaya R. (2020). Thermo-mechanical characterization of a bio-composite mortar reinforced with date palm fiber. J. Eng. Fibers Fabr..

[14] Menyhárd A., Menczel J.D., Abraham T. (2020). Polypropylene fibers. Thermal Analysis of Textiles and Fibers.

[15] Hsie M., Tu C., Song P. (2008). Mechanical properties of polypropylene hybrid fiber-reinforced concrete. Mater. Sci. Eng. A.

[16] Ali-Boucetta T., Ayat A., Laifa W., Behim M. (2021). Treatment of date palm fibres mesh: Influence on the rheological and mechanical properties of fibre-cement composites. Constr. Build. Mater..

[17] Qin Y., Zhang X., Chai J., Xu Z., Li S. (2019). Experimental study of compressive behavior of polypropylene-fiber-reinforced and polypropylene-fiber-fabric-reinforced concrete. Constr. Build. Mater..

[18] Bentegri I., Boukendakdji O., Kadri E., Ngo T., Soualhi H. (2020). Rheological and tribological behaviors of polypropylene fiber reinforced concrete. Constr. Build. Mater..

[19] Yong Z.C., Yew M.K., Yew M.C., Beh J.H., Lee F.W., Lim S.K., Saw L.H. (2024). Utilizing bio-based and industrial waste aggregates to improve mechanical properties and thermal insulation in lightweight foamed macro polypropylene fibre-reinforced concrete. J. Build. Eng..

[20] Leong G.W., Mo K.H., Loh Z.P., Ibrahim Z. (2020). Mechanical properties and drying shrinkage of lightweight cementitious composite incorporating perlite microspheres and polypropylene fibers. Constr. Build. Mater..

[21] Yuan Z., Jia Y. (2021). Mechanical properties and microstructure of glass fiber and polypropylene fiber reinforced concrete: An experimental study. Constr. Build. Mater..

[22] Razmi A., Mirsayar M. (2017). On the mixed mode I/II fracture properties of jute fiber-reinforced concrete. Constr. Build. Mater..

[23] Hasan R., Sobuz M.H.R., Akid A.S.M., Awall M.R., Houda M., Saha A., Meraz M.M., Islam M.S., Sutan N.M. (2023). Eco-friendly self-consolidating concrete production with reinforcing jute fiber. J. Build. Eng..

[24] Veerappan P., Mani I., John A., Madhavan H. (2024). Experimental studies of coir and jute-fiber reinforced concrete with M-sand. MatériaJa.

[25] Nawab M.S., Ali T., Qureshi M.Z., Zaid O., Kahla N.B., Sun Y., Anwar N., Ajwad A. (2023). A study on improving the performance of cement-based mortar with silica fume, metakaolin, and coconut fibers. Case Stud. Constr. Mater..

[26] Vélez E., Rodríguez R., Yanchapanta Gómez N.B., Mora E.D., Hernández L., Albuja-Sánchez J., Calvo M.I. (2022). Coconut-fiber composite concrete: Assessment of mechanical performance and environmental benefits. Fibers.

[27] Kiamahalleh M.V., Gholampour A., Ngo T.D., Ozbakkaloglu T. (2024). Mechanical, durability and microstructural properties of waste-based concrete reinforced with sugarcane fiber. Structures.

[28] Abdulkareem M., Ayeronfe F., Jassam T.M., AlAteah A.H., Al-Sodani K.A.A., Al-Tholaia M.M., Yam H., Ganiyu A., Alih S.C. (2024). Compressive and flexural strengths of bio-recycled concrete incorporated with kenaf fibre. J. Nat. Fibers.

[29] Antwi-Afari B.A., Mutuku R., Kabubo C., Mwero J., Mengo W.K. (2024). Influence of fiber treatment methods on the mechanical properties of high strength concrete reinforced with sisal fibers. Heliyon.

[30] Wang R., Qiao Z., Deng X., Shen X., Yang Y., Wang P., Zhang J. (2025). Experimental Investigation on Freeze–Thaw Durability of Polyacrylonitrile Fiber-Reinforced Recycled Concrete. Materials.

[31] Zhao X., Cai L., Ji X., Zeng W., Liu J. (2022). Mechanical properties of polyethylene fiber reinforced ultra high performance concrete (UHPC). Materials.

[32] Flores Nicolás A., Menchaca Campos E.C., Flores Nicolás M., Martínez González J.J., González Noriega O.A., Uruchurtu Chavarín J. (2024). Influence of Recycled High-Density Polyethylene Fibers on the Mechanical and Electrochemical Properties of Reinforced Concrete. Fibers.

[33] Sridhar M., Kumar M.V., Nagaprasad N., Bhagat S.K., Ramaswamy K. (2025). Microstructural and statistical analysis on mechanical performance of novel flattened end nylon fibre reinforced concrete. Sci. Rep..

[34] Ahmad J., Zaid O., Aslam F., Martínez-García R., Alharthi Y.M., Hechmi EI Ouni M., Tufail R.F., Sharaky I.A. (2021). Mechanical properties and durability assessment of nylon fiber reinforced self-compacting concrete. J. Eng. Fibers Fabr..

[35] Chandrasekhar C., Ransinchung RN G., Rajkumar G. (2025). Development and performance analysis of PVA-polyester fiber reinforced cementitious composites incorporating manufactured sand. Eur. J. Environ. Civ. Eng..

[36] Alawar A., Hamed A.M., Al-Kaabi K. (2009). Characterization of treated date palm tree fiber as composite reinforcement. Compos. Part B Eng..

[37] Adamu M., Marouf M.L., Ibrahim Y.E., Ahmed O.S., Alanazi H., Marouf A.L. (2022). Modeling and optimization of the mechanical properties of date fiber reinforced concrete containing silica fume using response surface methodology. Case Stud. Constr. Mater..

[38] Ali A., Hussain Z., Akbar M., Elahi A., Bhatti S., Imran M., Zhang P., Leslie Ndam N. (2022). Influence of Marble Powder and Polypropylene Fibers on the Strength and Durability Properties of Self-Compacting Concrete (SCC). Adv. Mater. Sci. Eng..

[39] Derdour D., Behim M., Benzerara M. (2023). Effect of date palm and polypropylene fibers on the characteristics of self-compacting concretes: Comparative study. Frat. Ed Integrità Strutt..

[40] Ahmad J., Burduhos-Nergis D.D., Arbili M.M., Alogla S.M., Majdi A., Deifalla A.F. (2022). A review on failure modes and cracking behaviors of polypropylene fibers reinforced concrete. Buildings.

[41] Osman K.M., ALyamany M.A., Tawab A., Thabet A. (2020). Effect of Using Natural and Polypropylene Fibers on Fresh and Hardened Concrete Properties. J. Mech. Civ. Eng..

[42] Abdelmajeed Labib W. Utilisation of date palm fibres in cement-based composites: A feasibility study. Proceedings of the IOP Conference Series: Materials Science And Engineering.

[43] Al-Sulaiman F.A. (2002). Mechanical properties of date palm fiber reinforced composites. Appl. Compos. Mater..

[44] Labib W.A. (2022). Plant-based fibres in cement composites: A conceptual framework. J. Eng. Fibers Fabr..

[45] Labib W.A. (2020). Evaluation of hybrid fibre-reinforced concrete slabs in terms of punching shear. Constr. Build. Mater..

[46] Aît-Mokhtar A., Belarbi R., Benboudjema F., Burlion N., Capra B., Carcassès M., Colliat J.-B., Cussigh F., Deby F., Jacquemot F. (2013). Experimental investigation of the variability of concrete durability properties. Cem. Concr. Res..

[47] Amran M., Al-Fakih A., Chu S., Fediuk R., Haruna S., Azevedo A., Vatin N. (2021). Long-term durability properties of geopolymer concrete: An in-depth review. Case Stud. Constr. Mater..

[48] (2022). Standard Specification for Portland Cement.

[49] (2023). Standard Specification for Concrete Aggregates.

[50] (2006). Fibres for Concrete—Polymer Fibres. Definitions, Specifications and Conformity.

[51] (2015). Standard Specification for Fiber-Reinforced Concrete.

[52] (1991). Standard Practice for Selecting Proportions for Normal, Heavyweight, and Mass Concrete.

[53] (2018). Standard Practice for Making and Curing Concrete Test Specimens in the Laboratory.

[54] (2012). Standard Test Method for Slump of Hydraulic-Cement Concrete.

[55] (2017). Standard Test Method for Density (Unit Weight), Yield, and Air Content (Gravimetric) of Concrete.

[56] (2009). Testing Hardened Concrete. Compressive Strength of Test Specimens.

[57] (2009). Testing Hardened Concrete-Tensile Splitting Strength Of Test Specimens.

[58] (2016). Standard Test Method for Flexural Strength of Concrete (Using Simple Beam With Center-Point Loading).

[59] (2021). Standard Test Method for Density, Absorption, and Voids in Hardened Concrete.

[60] (2022). Standard Guide for Examination of Hardened Concrete Using Scanning Electron Microscopy.

[61] (2003). Standard Test Method for Determining Pore Volume Distribution of Catalysts by Mercury Intrusion Porosimetry.

[62] Acosta-Calderon S., Gordillo-Silva P., García-Troncoso N., Bompa D.V., Flores-Rada J. (2022). Comparative evaluation of sisal and polypropylene fiber reinforced concrete properties. Fibers.

[63] Rachedi M., Kriker A. (2020). Thermal properties of plaster reinforced with date palm fibers. Civ. Environ. Eng..

[64] Vantadori S., Carpinteri A., Zanichelli A. (2019). Lightweight construction materials: Mortar reinforced with date-palm mesh fibres. Theor. Appl. Fract. Mech..

[65] Boumhaout M., Boukhattem L., Hamdi H., Benhamou B., Ait Nouh F. (2017). Thermomechanical characterization of a bio-composite building material: Mortar reinforced with date palm fibers mesh. Constr. Build. Mater..

[66] Adamu M., Ibrahim Y.E., Ahmed O.S., Drmosh Q.A. (2023). Mechanical performance of date palm fiber-reinforced concrete modified with nano-activated carbon. Nanotechnol. Rev..

[67] Abaeian R., Behbahani H.P., Moslem S.J. (2018). Effects of high temperatures on mechanical behavior of high strength concrete reinforced with high performance synthetic macro polypropylene (HPP) fibres. Constr. Build. Mater..

[68] Althoey F., Hakeem I.Y., Hosen M.A., Qaidi S., Isleem H.F., Hadidi H., Shahapurkar K., Ahmad J., Ali E. (2022). Behavior of concrete reinforced with date palm fibers. Materials.

[69] Blazy J., Blazy R. (2021). Polypropylene fiber reinforced concrete and its application in creating architectural forms of public spaces. Case Stud. Constr. Mater..

[70] Ozerkan N.G., Ahsan B., Mansour S., Iyengar S.R. (2013). Mechanical performance and durability of treated palm fiber reinforced mortars. Int. J. Sustain. Built Environ..

[71] Benmansour N., Agoudjil B., Gherabli A., Kareche A., Boudenne A. (2014). Thermal and mechanical performance of natural mortar reinforced with date palm fibers for use as insulating materials in building. Energy Build..

[72] Fallah S., Nematzadeh M. (2017). Mechanical properties and durability of high-strength concrete containing macro-polymeric and polypropylene fibers with nano-silica and silica fume. Constr. Build. Mater..

[73] Hannawi K., Bian H., Prince-Agbodjan W., Raghavan B. (2016). Effect of different types of fibers on the microstructure and the mechanical behavior of ultra-high performance fiber-reinforced concretes. Compos. Part B Eng..

